# The anti‐metastatic effect of ginsenoside Rb2 in colorectal cancer in an EGFR/SOX2‐dependent manner

**DOI:** 10.1002/cam4.1800

**Published:** 2018-09-27

**Authors:** Lan Thi Hanh Phi, Yoseph Toni Wijaya, Ita Novita Sari, Ying‐Gui Yang, Yun Kyung Lee, Hyog Young Kwon

**Affiliations:** ^1^ Soonchunhyang Institute of Medi‐bio Science (SIMS) Soonchunhyang University Cheonan Korea

**Keywords:** colorectal cancer, EGFR, EMT, ginsenoside Rb2, metastasis

## Abstract

Ginsenoside Rb2, a saponin from *Panax ginseng*, has been shown to have many functions. However, the effect of ginsenoside Rb2 on the metastasis of colorectal cancer (CRC) remains unknown. CRC cell lines HT29 and SW620 were used to determine the effects of ginsenoside Rb2 on the colony‐forming, migration, invasion, and wound‐healing abilities of CRC cells in vitro. Further, ginsenoside Rb2 was given intraperitoneally at 5 mg/kg of mouse body weight to check its effect on the metastasis of CRC cells in vivo. Ginsenoside Rb2 decreased colony‐forming ability, migration, invasion, and wound healing of CRC cells in vitro, although it did not affect cell proliferation. As a possible mechanism, we found that ginsenoside Rb2 down‐regulated the expression of stemness and Epithelial–mesenchymal transition (EMT)‐related genes via the EGFR/SOX2 signaling axis; these were partially rescued by either exogenous EGF treatment or ectopic expression of SOX2. More importantly, ginsenoside Rb2 significantly reduced the number of metastatic nodules in the livers, lungs, and kidneys in a mouse model of metastasis. These results suggest that ginsenoside Rb2 could be used to treat the metastasis of CRC therapeutically or as a supplement.

## 1 INTRODUCTION

Colorectal cancer (CRC) is the third most common cancer diagnosed in both men and women, and the second leading cause of cancer‐related deaths in the world. The development of CRC occurs through an ordered series of events called “adenoma‐carcinoma sequence”.^1^ This multifactorial process is accompanied by specific genetic changes, including both inactivation of the proto‐oncogenes adenomatous polyposis coli (APC) and P53, as well as activating mutations in the KRAS proto‐oncogene.^2^ Many options for CRC treatment are available, such as surgery, chemotherapy, radiation therapy, immunotherapy, and nutritional‐supplement therapy, but the success rates are not very promising.^3^ Metastasis, an important event contributing to drug resistance and cancer relapse, involves a complex series of steps in which cancer cells leave the original tumor site and spread to distant places in the body, through the blood or the lymph system. This has been the leading reason for the death of most cancer patients.^4^ More than 50% of CRC patients will develop liver metastasis during their lifespan, and almost half of the patients that undergo resection for primary colorectal cancer will eventually develop metachronous liver metastasis. If this occurs, survival time is no longer than three years, in spite of the improvements in current chemotherapies and biological agents .^5^

Ginsenosides, also known as triterpene saponins, are the pharmacologically active ingredients in ginseng extract.^6^ Ginseng is reported to have a variety of therapeutic and pharmacological uses, including anti‐hyperglycemic, anticancer, and neuroprotective activities.^7^ More than 40 ginsenosides have been identified, and the known ginsenosides can be classified into two structural categories: the 20(S)‐protopanaxadiol (PPD) (Rb1, Rb2, Rb3, Rc, Rb2, Rg3, Rh2, Rs1) and the 20(S)‐protopanaxatriol (PPT) (Re, Rf, Rg1, Rg2, Rh1); the only difference between PPTs and PPDs is the presence of a carboxyl group at the C‐6 position of PPDs.^6^ Ginsenoside Rb2 is one of the key active components in ginseng and has been demonstrated to have various potential pharmacological effects. Previous studies have revealed the anti‐carcinogenic activity of ginsenoside Rb2 on human CRC cells^8^ and lung cancer.^9^ However, it still remains unknown whether ginsenoside Rb2 inhibits metastasis of CRC. Here, we addressed these questions and demonstrated that ginsenoside Rb2 regulates epidermal growth factor receptor (EGFR), as well as its downstream targets, leading to inhibition of CRC metastasis.

## 2 MATERIALS AND METHODS

### 2.1 Cell lines

Human colorectal cancer cell lines HT29 and SW620 were purchased from the Korean Cell Line Bank (KCLB). HT29 and SW620 cells were grown in RPMI‐1640 medium (Corning, New York, USA) supplemented with 10% Fetal bovine serum FBS (Corning), 1% MEM essential amino acids (Corning), and 1% penicillin/streptomycin (Gibco, Waltham, Massachusetts, USA) at 37°C in a humidified atmosphere containing 5% CO_2_. To culture cells in stem cell selective media, we used DMEM/F12 medium in the absence of serum supplemented with 1% N2 supplement (Gibco, Waltham, Massachusetts, USA), 20 ng/mL epidermal growth factor EGF (Invitrogen, Waltham, Massachusetts, USA), and 20 ng/mL basic fibroblast growth factor bFGF (Gibco, Waltham, Massachusetts, USA) and 1% penicillin/streptomycin (Gibco, Waltham, Massachusetts, USA) using ultralow attachment plate (Corning).

### 2.2 Ginsenoside Rb2 preparation

Ginsenoside Rb2 was obtained from Korean Ginseng Corporation and Ambo Institute (Korea). It was dissolved at a concentration of 20 mmol/L in DMSO as a stock solution and stored in aliquots at −20°C.

### 2.3 Retroviral constructs and transfection

The open reading frame (ORF) of SOX2 (Forward, 5'‐GCCG GAATTC ATGTACAACATGATGGAGACGGAG‐3' and reverse, 5'‐GCCGCTCGAGTCACATGTGTGAGAGGGG‐3') and SNAIL (Forward, 5'‐ AGTCCAGAATTCATGCCGCGCTCTTTCCTCGTCAGGA −3' and reverse, 5'‐ AGTCCACTCGAGTCAGCGGGGACATCCTGAGCAGCCG −3') was amplified and cloned into MSCV‐hCD2 and MSCV‐IRES‐GFP vector, respectively. Virus was produced in 293 T cells transfected with viral constructs along with Gag/pol and VSVG constructs using the iN‐fect^TM^ in vitro transfection reagents (iNtRON, Seongnam, Korea) following the manufacturer's protocol.

### 2.4 RNA extraction and real‐time PCR

RNA was isolated using Ribospin II or Hybrid R (Gene All, Seoul, Korea) and converted to cDNA using ReverTra Ace^®^ qPCR Kit (TOYOBO, Osaka, Japan) according to the manufacturer's instructions. To determine the level of gene expression, qPCR was performed using the TOPreal™ qPCR 2X PreMIX (Enzynomics, Korea). Primer sequences for RT‐qPCR are shown in Table S1.

### 2.5 Western blotting analysis

Cell lysates were harvested using cell lysis buffer, and an equal amount of each protein extract was resolved using 10% polyacrylamide gel and electro‐transferred onto 0.45‐μm hybridization nitrocellulose filter (HATF) membrane (Millipore, USA). Membranes were immunoblotted with goat polyclonal anti‐ACTIN antibody, rabbit monoclonal anti‐SNAIL antibody, rabbit monoclonal anti‐SOX2 antibody, rabbit polyclonal anti‐EGFR antibody, rabbit polyclonal anti‐pEGFR antibody, rabbit polyclonal anti‐AKT antibody, and rabbit polyclonal anti‐pAkt antibody (Cell Signaling, Danvers, Massachusetts, USA) overnight at 4°C. Membranes were immunobloted with either HRP‐conjugated anti‐rabbit immunoglobulin (Cell Signaling, Danvers, Massachusetts, USA) or HRP‐linked anti‐goat immunoglobulin (Santa Cruz Biotechnology, Dallas, Texas, USA) for 1 hour at room temperature. The protein signal was detected by enhanced chemiluminescence (Thermo, Waltham, Massachusetts, USA) using the Amersham Imager 600 (GE Healthcare Life Sciences, Chicago, Illinois, USA).

### 2.6 Cell proliferation assay (MTT assay)

Cell proliferation was examined using Cell Proliferation Kit I MTT assay (Roche, Basel, Switzerland). Briefly, 5 × 10^3 ^colorectal cancer cells were seeded and incubated for an additional 96 hours in the presence or absence of ginsenoside Rb2. Cells were then incubated in 5 mg/mL of MTT solution for 4 hours, followed by solubilization with 100 µL solubilization solution (10% SDS in 0.01 mol/L HCl) overnight. Absorbance was read at 575 and 650 nm using a plate reader.

### 2.7 Quantitation of apoptotic cells (Annexin V assay)

The Annexin V assay was carried out using the eBioscience™ Annexin V Apoptosis Detection Kit eFluor™ 450 (eBioscience Inc., San Diego, CA, USA). The cells were seeded in 24‐well plate with 1 × 10^5^ cells/well and were incubated overnight. These cells were then treated with ginsenoside Rb2 at three different concentrations (10, 50, and 100 µmol/L) for 48 hours. The cells were harvested, subsequently stained with Annexin V and 7AAD, and then analyzed using flow cytometer.

### 2.8 Soft agar colony‐forming assay

In preparation for the assay, 1% agarose in complete medium was coated onto 24‐well plates and allowed to cool for 30 minutes at room temperature. Cells suspension (2 × 10^3 ^cells/well) and ginsenoside were mixed with 0.3% agarose in complete medium and plated on top of the 1% agarose base layer. Subsequently, complete medium was applied on top of the cell layer to avoid evaporation. The number and size of colonies were observed after 15 days.

### 2.9 Migration and invasion assay

After treated, cells were trypsinized and counted. Cell migration and invasion were analyzed in vitro using the transwell insert system (Corning) without coating or with coating by 20 μL of Matrigel (BD Biosciences, USA), respectively. The culture insert was attached on the bottom of a 24‐well plate, and 100 μL of serum‐free media containing 1 × 10^5^ cells was seeded into each well of the insert. Six hundred μL of media containing 10% FBS was added outside the transwell culture insert. Cells were incubated at 37°C for 18 and 24 hours in a humidified atmosphere with 5% CO2 for migration and invasion, respectively. Transwells were cleaned using cotton swap. The cells were fixed with 1% formaldehyde for 15 minutes, washed twice with Phosphate buffered saline (PBS), stained with 0.1% of crystal violet for 15 minutes, washed with distilled water, and then observed using a microscope (Leica, Wetzlar, Germany).

### 2.10 Wound‐healing assay

Cells were cultivated in 24‐well plate at a density of 1 × 10^5^ cells/well 24 hours before treatment. When the cell density reached more than 90% confluence, the medium was removed. A line was scratched using the end of a 200‐mL pipette tip (time 0 hour), and cells were washed twice with PBS to remove the loose cells. Cells were treated with or without ginsenoside in serum‐free media for 48 hours. Images of migrating cells were taken every 24 hours.

### 2.11 Gelatin zymography analysis

Cells were treated with ginsenoside in the media without serum, and the supernatant was collected to determine the activity of MMP2 after 96 hours. Samples were analyzed on SDS‐PAGE containing 0.1% gelatin. After electrophoresis, the gel was renatured two times with 2.5% Triton X‐100 for 30 minutes at room temperature, followed by washing with ddH^2^O. The gel was incubated in developing buffer (50 mmol/L Tris‐HCl pH 7.6, 50 mmol/L NaCl, 10 mmol/L CaCl^2^, 0.05% Brij 35) for 24 hours at 37°C. Gel staining was conducted for 1 hour at room temperature using Coomassie brilliant blue protein staining and destained using destaining solution (methanol:ddH^2^O:acetic acid = 5:4:1) at room temperature.

### 2.12 Flow cytometry

Using StemPro Accutase Cell Dissociation Reagent (Gibco, Waltham, Massachusetts, USA), the single cells were separated from the sphere after culturing. These single cells are then centrifuged to precipitate and washed with DPBS (Gibco, Waltham, Massachusetts, USA). To block unspecific Fc interaction, the cells were incubated with human Fc blocker in 100 μL of flow cytometry buffer (2% FBS in PBS) for 10 minutes on ice. Cells were then labeled with PE‐conjugated anti‐CD133, APC‐conjugated anti‐CD44 monoclonal antibody (mAb) (eBioscience) for further 30 minutes on ice. After incubating, cells were washed and were analyzed using flow cytometry. We also included the Fixable Viability Dye (FVD) (eBioscience, USA) for dying and dead cells exclusion.

### 2.13 Animal experiments

HT29 CRC cells were trypsinized and resuspended in PBS at a final concentration of 1.5 × 10^7 ^cells/ml. 1.5 × 10^6 ^cells were injected intravenously to eight‐week‐old female NSG mice (NOD.Cg‐*Prkdc^scid^ Il2rg^tm1Wjl^*/SzJ, The Jackson Laboratory). Following injection 48 hours, mice were randomly divided into two groups (control and ginsenoside Rb2 treatment) and were administrated (intraperitoneal injection) with 5 mg/kg/mice ginsenoside Rb2 or the equal volume of PBS three times per week. The mice were observed daily and sacrificed after 28 days. The number and weight of tumor nodules on the surface of the liver, lung, and spleen were counted, measured, and statistically analyzed. Slides with 4‐5 μm thick liver section were prepared, paraffin‐embedded and then stained with hematoxylin and eosin (H&E). All experimental protocols were approved by Soonchunhyang University Institutional Animal Care and Use Committee.

### 2.14 Statistical analysis

All experiments were independently performed at least three times. The results of RT‐qPCR, Western blot, gelatin zymography, migration, and invasion were analyzed with Student's *t* test. Differences were considered statistically significant at *P* < 0.05 (*) or highly significant at *P* < 0.01 (**).

## 3 RESULTS

### 3.1 Ginsenoside Rb2 inhibits the colony‐forming ability of CRC cells

**Figure 1:**
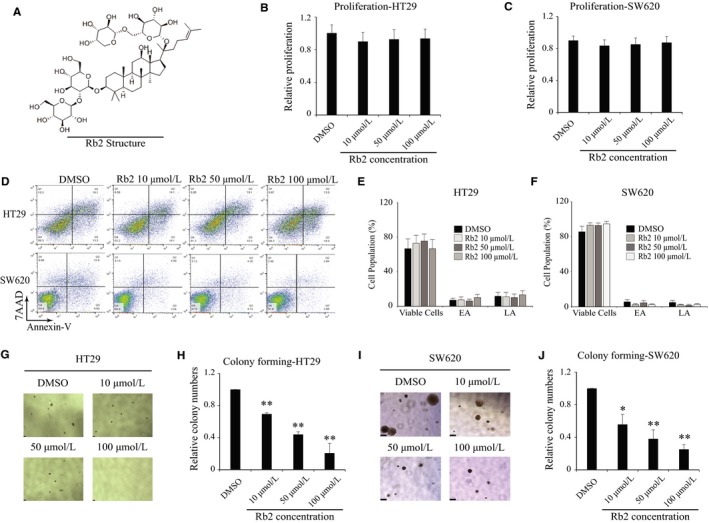
The colony‐forming ability of CRC cells is suppressed by ginsenoside Rb2. (A) The chemical structure of ginsenoside Rb2. (B and C) Both CRC cell lines HT29 and SW620 were seeded in 96‐well plate and treated with ginsenoside Rb2 with different concentrations. The MTT assay was used for measuring the cell proliferation after 72 h. (D‐F) HT29 and SW620 cells were seeded and treated with ginsenoside Rb2 for 48 h. The apoptotic effect of ginsenoside Rb2 was analyzed using Annexin V assay. EA and LA stand for early apoptosis and late apoptosis, respectively. A representative image was shown (D), and the data were presented as the mean ±SEM of two independent experiments (E and F). (G‐J) Both CRC cell lines HT29 and SW620 were seeded in complete media containing 0.3% of agarose with either DMSO or ginsenoside Rb2 at different concentrations. The number of colony was counted after incubating 15 d. (D and E) A representative image was shown. The statistical analysis is shown (*, *P* < 0.05; **, *P* < 0.01). The data are presented as the mean ±SEM of three independent experiments

The structure of ginsenoside Rb2 obtained from *Panax ginseng* is shown in Figure 1A. Firstly, we tested whether ginsenoside Rb2 has any inhibitory effect on the proliferation of CRC cell lines, such as HT29 and SW620. CRC cell lines were cultured together with ginsenoside Rb2 at different concentrations (0, 10, 50, 100 μmol/L) for 72 hours, and the proliferation was determined using MTT assay. The proliferation of CRC cell lines was not affected, even at 100 μmol/L of ginsenoside Rb2 (Figure 1B,C). To determine the apoptotic effects of ginsenoside Rb2 on CRC cells, both HT29 and SW620 cells were treated with ginsenoside Rb2 and apoptosis rates were determined using Annexin V assay. Consistent with MTT assay, Annexin V assay indicated that ginsenoside Rb2 treatment did not have any apoptotic effect in colorectal cancer cells compared to the control (Figure 1D‐F). These days, three‐dimensional (3D) culture is considered to reflect tumor microenvironment more accurately than two‐dimensional (2D) culture,^10^ making it an attractive model for the testing of anticancer drugs.^11^ Thus, we tested the effect of ginsenoside Rb2 on the colony‐forming ability of the CRC cell line, a technique widely used to evaluate the growth and drug sensitivity of cancer stem cells (CSCs) in 3D culture.^12^ Both HT29 and SW620 CRC cell lines were cultured in soft agar‐containing media, in the absence or presence of ginsenoside Rb2 for 15 days, and then the number of colonies was counted. Both the number and size of colonies formed in the presence of ginsenoside Rb2 were significantly reduced in a dose‐dependent manner, compared to the untreated group (HT29: 70%, 44%, and 21% at 10, 50, and 100 μmol/L, respectively; SW620: 56%, 38%, and 25% at 10, 50, and 100 μmol/L, respectively) (Figure 1G‐J). As colony‐forming ability is related to the characteristics of CSCs, we determined whether ginsenoside Rb2 influences the expression of CSC markers, such as CD133 and CD24, by flow cytometry.^13^, ^14^ The mean fluorescent intensity of cell surface markers CD133 and CD24 of SW620 cells was decreased by ginsenoside Rb2 treatment (Figure S1). These data indicate that ginsenoside Rb2 inhibits the colony‐forming ability and reduces the expression of CSC markers in CRC cell lines, thus a promising method to target CSCs in CRC.

### 3.2 Ginsenoside Rb2 inhibits the migration and invasion of CRC cells

**Figure 2:**
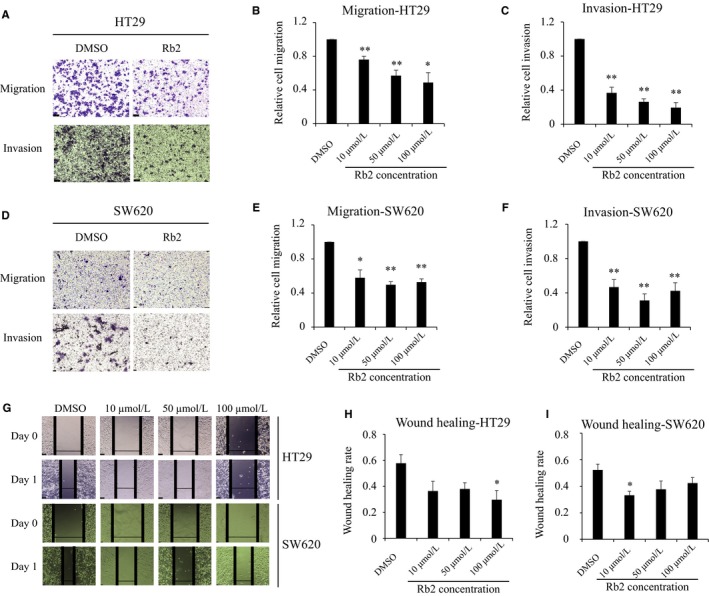
Ginsenoside Rb2 inhibits the mobility of CRC cells. (A‐F) The migration and invasion of HT29 (A‐C) and SW620 (D‐F) cells were examined in the presence of ginsenoside Rb2 at the concentration indicated using transwell insert system with non‐coated membrane and matrigel‐coated membrane, respectively. Representative images were shown (A and D). (G‐I) In wound‐healing assay, HT29 (G and H) and SW620 (G and I) cells were seeded and scratched in the middle. The cells were washed twice with PBS and treated with either DMSO or ginsenoside Rb2 for 24 h. The statistical analysis is shown (*, *P* < 0.05; **, *P* < 0.01). The data are presented as the mean ± SEM of three independent experiments

We then investigated whether ginsenoside Rb2 could inhibit the migration and invasion of CRCs, as cell motility is one of the essential mechanisms of cancer metastasis.^15^ To study the effects of ginsenoside Rb2 on cell migration, a transwell system with non‐coated inserts was utilized. Both HT29 and SW620 CRC cell lines were treated with ginsenoside Rb2 at the concentration indicated for 48 hours in the absence of serum, then incubated for 18 hours on a transwell insert. The cells on the inserts of transwell system were stained after washing, and then counted to evaluate the migration of cancer cells. Ginsenoside Rb2‐treated cancer cells migrated about 49%‐76% of control‐treated cells at the concentrations tested (HT29: 76%, 57%, and 49% at 10, 50, 100 μmol/L, respectively; SW620: 58, 50, 53% at 10, 50, and 100 μmol/L, respectively) (Figure 2A, B, D and E). We also determined whether ginsenoside Rb2 affects the invasion of cancer cells using a matrigel‐coated insert of the transwell system. CRC cell lines treated with ginsenoside Rb2 for 48 hours, in the absence of serum, were tested for their ability to invade a matrigel‐coated insert for 24 hours. Ginsenoside Rb2 significantly decreased the invasion of CRC cells by 53%‐81% of control‐treated cells at the concentrations tested (HT29: 63%, 74%, and 81% at 10, 50, and 100 μmol/L, respectively; SW620: 53%, 69%, and 58% at 10, 50, and 100 μmol/L, respectively) (Figure 2A, C, D and F). In agreement with the transwell assay, a scratch‐induced wound‐healing assay also showed that wound healing was significantly delayed in the presence of ginsenoside Rb2 (Figure 2G‐I). These results indicate that ginsenoside Rb2 significantly inhibits the mobility of CRC cell lines.

### 3.3 Ginsenoside Rb2 inhibits CSC and EMT signature of CRC cells

**Figure 3:**
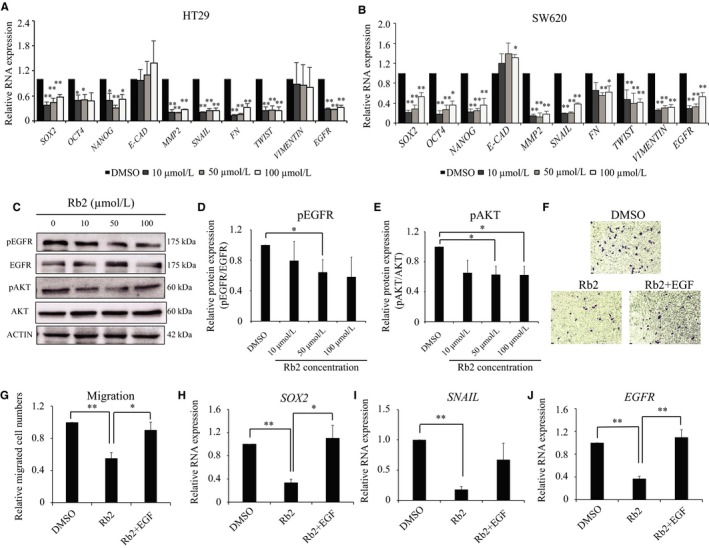
Ginsenoside Rb2 inhibits the CSC‐like properties and mobility of CRC cells via EGFR signaling. HT29 (A) and SW620 (B) cells were treated with ginsenoside Rb2, and the expression levels of CSC and EMT markers were determined by RT‐qPCR. (C‐E) HT29 cells were treated with ginsenoside Rb2 and EGF at different concentrations for 24 h. Then, the phosphorylation level and total protein level of EGFR (C and D) and AKT (C and E) were determined by immunoblots. (F‐J) HT29 cells were treated with either DMSO, ginsenoside Rb2 alone or ginsenoside Rb2 together with 200 ng/mL of EGF in serum‐free media for 2 d. Then, the cells were seeded for migration assay (F and G) or RNA was extracted to determine the expression of *SOX2* (H), *SNAIL* (I), and *EGFR* (J) by RT‐qPCR. The statistical analysis is shown (*, *P* < 0.05; **, *P* < 0.01). The data are presented as the mean ± SEM of three independent experiments

We found that ginsenoside Rb2 inhibits both colony‐forming ability and migratory ability, which are related to CSC and epithelial‐mesenchymal transition (EMT) (Figures 1 and 2). Thus, we examined whether ginsenoside Rb2 influences the signatures of CSC and EMT in CRC as possible mechanisms. To test this, both HT29 and SW620 CRC cell lines were treated with ginsenoside Rb2 at 10‐100 μmol/L for 4 days. Then, RNA was isolated to determine the expression of CSC and EMT signatures by RT‐qPCR. As shown in Figure 3A,B, the CSC and EMT signatures were significantly dysregulated by the treatment of ginsenoside Rb2. Specifically, CSC signatures such as *SOX2*, *OCT4* and *NANOG* were significantly down‐regulated in the presence of ginsenoside Rb2. Also, mesenchymal signatures including *SNAIL, FIBRONECTIN *(*FN*), *TWIST*, *VIMENTIN,* and *MMP2* were down‐regulated by ginsenoside Rb2, whereas *E‐CAD* (epithelial signature) was upregulated (Figure 3A,B). Interestingly, we also found that *EGFR* expression also was down‐regulated by ginsenoside Rb2 (Figure 3A,B). It was shown that ECM degradation via the increased activity of proteolytic enzymes, including MMPs, can lead to cancer cell migration and metastasis.^16^ To investigate whether ginsenoside Rb2 inhibited the migration and invasion of colorectal cancer cells through inhibition of the activity of MMP2, also known as a potential prognostic biomarker of colorectal cancer,^17^ we conducted a gelatin zymography assay with the culture supernatant, after treating HT29 cells with ginsenoside Rb2 at different concentrations for 4 days. Consistent with the RNA level of *MMP2* being reduced by ginsenoside Rb2 (Figure 3A,B), we found from the zymography assay that MMP2 enzymatic activity was significantly down‐regulated by ginsenoside Rb2 (Figure S2). These data suggest that ginsenoside Rb2 inhibits the mobility of CRCs via the regulation of CSC and EMT signatures.

### 3.4 EGFR‐AKT signaling pathway mediates the inhibitory effects of ginsenoside Rb2

Previously, by reverse docking, Park et al demonstrated that ginsenoside Rb2 strongly interacts with both wild‐type and mutant forms of EGFR at different residues, such as MET769, GYS773, and GLU734 or PRO794, ASP855, and LYS716.^18^ Moreover, increasing evidences suggest that the expression and phosphorylation of EGFR directly correlate with the poor prognosis and metastasis in CRC.^19^, ^20^, ^21^, ^22^, ^23^, ^24^ Thus, we tested whether the down‐regulation of CSC and EMT signature by ginsenoside Rb2 occurs through EGFR signaling. To test this, HT29 cells were treated with ginsenoside Rb2 together with epidermal growth factor (EGF) in stem cell selective media for 24 hours, and then the phosphorylation level of EGFR was determined by immunoblots. As shown in Figure 3B, the level of pEGFR (pEGFR/tEGFR) was significantly reduced at 50 μmol/L of ginsenoside Rb2, compared to control (Figure 3C,D). The AKT pathway is also well‐known as a downstream target of EGFR signaling by the generation of phosphatidylinositol‐3,4,5‐trisphosphate (PIP3).^25^, ^26^ Thus, we then examined whether AKT is also influenced by ginsenoside Rb2. Consistently, pAKT level was also significantly decreased by ginsenoside Rb2 at both 50 μmol/L and 100 μmol/L, compared to control (Figure 3C,E). As we found that migratory ability was reduced by ginsenoside Rb2 and that EGFR/AKT signaling was impaired by the treatment of ginsenoside Rb2, we tested whether the reduced migration by ginsenoside Rb2 could be reversed by excess EGF treatment. HT29 cancer cells were treated with either DMSO, 50 μmol/L of ginsenoside Rb2 alone or 50 μmol/L of ginsenoside Rb2 together with EGF. Then, the migration of cancer cells was tested. Ginsenoside Rb2 inhibited the migration of cancer cells, as shown before, and was reversed by the treatment of EGF (Figure 3F,G). We found that reduced expression of EGFR downstream signaling pathways caused by ginsenoside Rb2 treatment, including *SOX2*, *SNAIL,* and *EGFR*, was partially rescued by EGF treatment (Figure 3H‐J). These results suggest that ginsenoside Rb2 inhibits the CSC‐like properties and EMT signaling via blockade of EGFR/AKT signaling pathway.

### 3.5 Effects of ginsenoside Rb2 on CRC are partly rescued by ectopic expression of SOX2 or SNAIL

**Figure 4:**
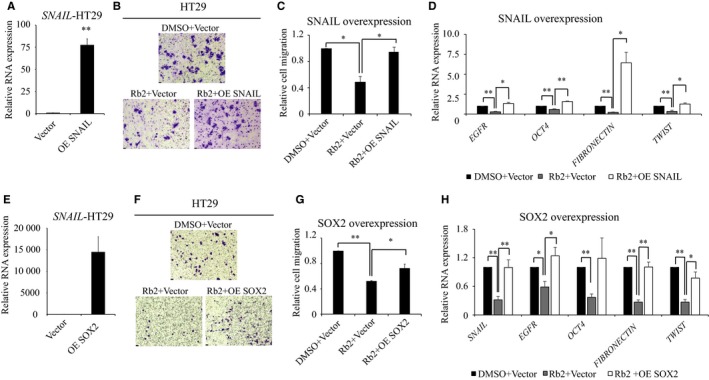
The ectopic expression of SOX2 or SNAIL partly rescues the inhibitory effect of ginsenoside Rb2 on CRC cells. (A‐D) HT29 cells transduced with either vector or overexpression of SNAIL were treated with either DMSO or 50 μmol/L of ginsenoside Rb2. *SNAIL* overexpression was confirmed by RT‐qPCR (A). The transduced cells were tested for migration (B and C) or RNA was isolated to determine the expression levels of target genes (D). (E‐H) HT29 cells transduced with either vector or overexpression of SOX2 were treated with either DMSO or 50 μmol/L of ginsenoside Rb2. *SOX2* overexpression was confirmed by RT‐qPCR (E). The transduced cells were tested for migration (F and G) or RNA was isolated to determine the expression levels of target genes (H). The statistical analysis is shown (*, *P* < 0.05; **, *P* < 0.01). The data are presented as the mean ± SEM of three independent experiments

As we found that ginsenoside Rb2 significantly inhibited the expression of SOX2 and SNAIL, and that both the expression of *SOX2 *and *SNAIL* were partly reverted to control levels by co‐treatment of ginsenoside Rb2 and EGF simultaneously, we hypothesized that the inhibition of both CSC‐like properties and EMT by ginsenoside Rb2 is via SOX2 and SNAIL, and that SOX2 and SNAIL may rescue the defects caused by ginsenoside Rb2. To test this, we ectopically expressed SOX2 or SNAIL, using a retroviral system, and determined their contribution to the migration ability of HT29 cells. The cells transduced with either vector, SOX2, or SNAIL overexpression constructs were treated with 50 μmol/L of ginsenoside Rb2, and the migration of the cells was determined. To begin with, we confirmed the overexpression of *SOX2* and *SNAIL* by RT‐qPCR (Figure 4A,E). The cells treated with ginsenoside Rb2 only migrated 50% compared to that of control cells, whereas those with SOX2 and SNAIL overexpression, together with ginsenoside Rb2, migrated 80% and 95% compared to that of control cells, respectively (Figure 4B, C, F, G and Figure S3). Furthermore, at the molecular level, we found that ectopic expression of either SOX2 or SNAIL rescued the expression of key signaling molecules implicated in CSCs and EMT, such as *EGFR*, *OCT4*, *FN*, and *TWIST* that were down‐regulated by ginsenoside Rb2 (Figure 4D,H). These results suggest that ginsenoside Rb2 inhibits the CSC‐like properties and EMT signature of CRC cells through SOX2 and SNAIL.

### 3.6 Ginsenoside Rb2 efficiently inhibits the metastasis of a CRC cells in vivo

**Figure 5:**
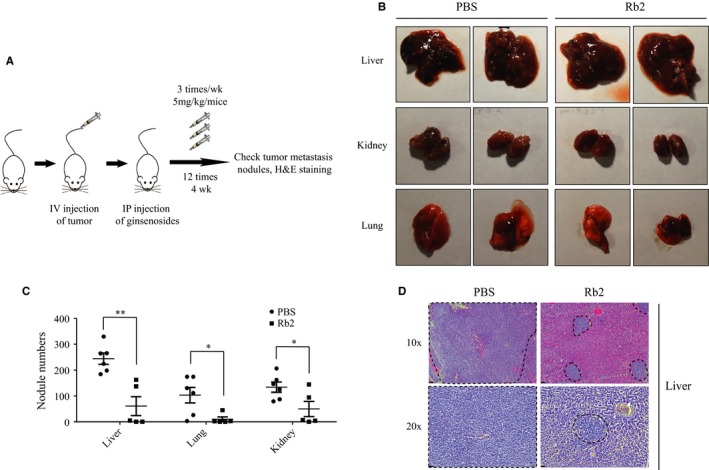
The metastasis of CRC cells is inhibited by ginsenoside Rb2 in a mouse model of metastasis. A, The schematic diagram of the experiment. Mouse metastasis model generated by intravenous injection of HT29 cells were treated with PBS (control) and ginsenoside Rb2 (5 mg/kg body weight) three times/wk for 4 wk. B, A representative image of the liver, lung, and kidney. C, Numbers of tumor metastasis nodules in the liver, lung, and kidney tissues were presented (PBS, n = 6; Rb2, n = 5). D, Liver segments stained with HE were shown. The dotted lines indicated the area of tumor nodules. The statistical analysis of nodule number is shown (*, *P* < 0.05; **, *P* < 0.01). The data are presented as the mean ± SEM

The aforementioned in vitro experiments prompted us to demonstrate the effects of ginsenoside Rb2 in tumor metastasis in vivo. Thus, by using a mouse metastasis model, we intravenously injected HT29 cells into immunocompromised Nod Scid Gamma (NSG) mice and determined whether ginsenoside Rb2 inhibits tumor metastasis in vivo. After 2 days of injection, we started treating the mice, with either ginsenoside Rb2 at 5 mg/kg per mice or PBS as control, three times per week by intraperitoneal injection. After 4 weeks, we sacrificed the mice and checked the number of metastatic nodules from the liver, lung, and kidney (Figure 5A). As shown in Figure 5B,C, ginsenoside Rb2 significantly reduced the number of nodules from each organ tested, compared to control (Liver: 244 vs 61; Lung: 103 vs 10; Kidney: 134 vs 50, *P* < 0.05). Consistently, histopathological H&E staining of various liver sections revealed that the sections from ginsenoside Rb2‐treated mice displayed a significantly smaller number and size of tumor nodules (Figure 5D). Thus, these data indicate that ginsenoside Rb2 treatment inhibits the metastatic ability of CRC cells in an in vivo mouse model of metastasis.

## 4 DISCUSSION

Among complementary and alternative medicine (CAM) used widely to treat a variety of health conditions, such as cancer,^27^ many traditional herbal medicines including ginseng have been used for therapeutic purposes.^28^ Previous studies indicated that ginsenoside Rb2, a major biologically active saponin of ginseng, has anticancer properties in several cancers, such as CRC and lung cancer.^8^, ^9^ In the present study, we showed that ginsenoside Rb2 suppressed CSC‐like properties, and migration and invasion of CRC cells in vitro. More importantly, ginsenoside Rb2 remarkably reduced the metastasis of CRC cells to the liver, lung, and kidney in vivo, in a mouse metastasis model. These results therefore indicate that ginsenoside Rb2 may be a promising therapeutic medication for the treatment of CRC metastasis.

EGFR, an important receptor tyrosine kinase (RTK), is a prognostic marker of CRC and significantlyassociated with TNM (tumor – node‐ metastasis) stage T3.^21^, ^23^, ^24^ Thus, several molecules targeting EGFR have been developed; gefitinib, erlotinib, afatinib, and icotinib for lung cancer^29^, ^30^ and cetuximab and panitumumab for CRC.^31^, ^32^ However, the efficacies of these molecules are not so high, or even ineffective against mutant forms of EGFR.^33^, ^34^ In a previous study on potential targets of ginsenosides, using a reverse docking assay, it was found that ginseng saponins, such as ginsenoside Rb2, Ro, and R2, interact with both wild‐type EGFR and mutant forms of EGFR with high binding affinity.^18^ Similarly, we also found that the inhibitory effect of Rb2 on CRC cells is through EGFR and its downstream signaling, SOX2, and SNAIL. We indicated that EGFR signaling was suppressed by ginsenoside Rb2 and was partially rescued by ectopic expression of SOX2 and SNAIL. Interestingly, it has been shown that activation of EGFR induces SOX2 expression, and reciprocally, SOX2 binds to the EGFR promoter, increasing the EGFR expression level, and thus, forming a positive feedback relationship between EGFR and SOX2.^35^, ^36^ Further, overexpression of SNAIL results from the activation of EGFR, which is most likely regulated by the p38 MAPK, ERK1/2, and the AKT/GSK‐3b pathways.^37^ Interestingly, SOX2 and SNAIL have been shown to play an essential role in the regulation of self‐renewal, expansion of CSCs, and metastasis in several cancers, including colorectal cancer.^38^, ^39^, ^40^ Thus, the EGFR/SOX2 signaling axis is an essential pathway in CRC progression and should be targeted for better therapeutic outcomes. Furthermore, as the mutant forms of EGFR tend to be resistant to the aforementioned therapeutics, we suggest that ginsenoside Rb2 could be used alternatively or as an adjuvant for the treatment of abnormal EGFR function.

To our knowledge, we report here for the first time that ginsenoside Rb2 inhibits CSC‐like properties and EMT of CRC cells, resulting in the suppression of the metastasis of CRC cells in vivo. Thus, we propose that ginsenoside Rb2 may be a plausible candidate that could be used to treat the CRC metastasis.

## CONFLICT OF INTEREST

The authors declare no conflict of interest.
