# Supplementary material for: The anti‐metastatic effect of ginsenoside Rb2 in colorectal cancer in an EGFR/SOX2‐dependent manner
Source: Cancer Med. 2018 Sep 27;7(11):5621–31. doi: 10.1002/cam4.1800 (PMC6246932; doi:10.1002/cam4.1800)
Supplement: Supplementary file 4 [file CAM4-7-5621-s004.docx]

**Supplementary Table 1. Primers used for RT-qPCR**

| B2M-F | TGAAGCTGACAGCATTCGG |
| --- | --- |
| B2M-R | CTGCTGGATGACGTGAGTAAA |
| EGFR-F | GCCAAGGCACGAGTAACAAGC |
| EGFR-R | AGGGCAATGAGGACATAACC |
| SNAI1-F | CTGGGTGCCCTCAAGATGCA |
| SNAI1-R | CCGGACATGGCCTTGTAGCA |
| Sox2-F | CCTTCTTCATGAGCGTCTTG |
| Sox2-R | GATGCACAACTCGGAGATC |
| Nanog-F | AGAAGGCCTCAGCACCTA |
| Nanog-R | GGCCTGATTGTTCCAGGA |
| Oct4-F | ACATCAAAGCTCTGCAGAAAGAAC |
| Oct4-R | CTGAATACCTTCCCAAATAGAACCC |
| MMP2-F | CGCTCAGATCCGTGGTGA |
| MMP2-R | CGCCAAATGAACCGGTCCTT |
| E-Cad-F | TTGCACCGGTCGACAAAGGAC |
| E-Cad-R | TGGATTCCAGAAACGGAGGCC |
| FN-F | GAGAATGGACCTGCAAGCCCA |
| FN-R | AGTGCAAGTGATGCGTCCGC |
| VIM-F | ACCCGCACCAACGAGAAGGT |
| VIM-R | ATTCTGCTGCTCCAGGAAGCG |
| TWIST1-F | TGCGGAAGATCATCCCCCG |
| TWIST1-R | GCTGCAGCTTGCCATCTTGGA |
